# Deep learning based retinal vessel segmentation and hypertensive retinopathy quantification using heterogeneous features cross-attention neural network

**DOI:** 10.3389/fmed.2024.1377479

**Published:** 2024-05-22

**Authors:** Xinghui Liu, Hongwen Tan, Wu Wang, Zhangrong Chen

**Affiliations:** ^1^School of Clinical Medicine, Guizhou Medical University, Guiyang, China; ^2^Department of Cardiovascular Medicine, Guizhou Provincial People's Hospital, Guiyang, China; ^3^Electrical Engineering College, Guizhou University, Guiyang, China; ^4^Department of Cardiovascular Medicine, The Affiliated Hospital of Guizhou Medical University, Guiyang, China

**Keywords:** retinal vessel segmentation, hypertensive retinopathy quantification, deep learning, cross-attention network, color fundus images

## Abstract

Retinal vessels play a pivotal role as biomarkers in the detection of retinal diseases, including hypertensive retinopathy. The manual identification of these retinal vessels is both resource-intensive and time-consuming. The fidelity of vessel segmentation in automated methods directly depends on the fundus images' quality. In instances of sub-optimal image quality, applying deep learning-based methodologies emerges as a more effective approach for precise segmentation. We propose a heterogeneous neural network combining the benefit of local semantic information extraction of convolutional neural network and long-range spatial features mining of transformer network structures. Such cross-attention network structure boosts the model's ability to tackle vessel structures in the retinal images. Experiments on four publicly available datasets demonstrate our model's superior performance on vessel segmentation and the big potential of hypertensive retinopathy quantification.

## 1 Introduction

Hypertension (HT) is a chronic ailment posing a profound menace to human wellbeing, manifesting in vascular alterations (1). Its substantial contribution to the global prevalence and fatality rates of cardiovascular diseases (CVD) cannot be overstated. The escalated incidence and mortality rates are not solely attributable to HT's correlation with CVD but also to the ramifications of hypertension-mediated organ damage (HMOD). This encompasses structural and functional modifications across pivotal organs, including arteries, heart, brain, kidneys, vessels, and the retina, signifying preclinical or asymptomatic CVD (2, 3). HT management's principal aim remains to deter CVD incidence and mortality rates. Achieving this goal mandates meticulous adherence to HT guidelines, emphasizing precise blood pressure monitoring and evaluating target organ damage (4). Consequently, the early identification of HT-mediated organ damage emerges as a pivotal concern.

**Figure 1:**
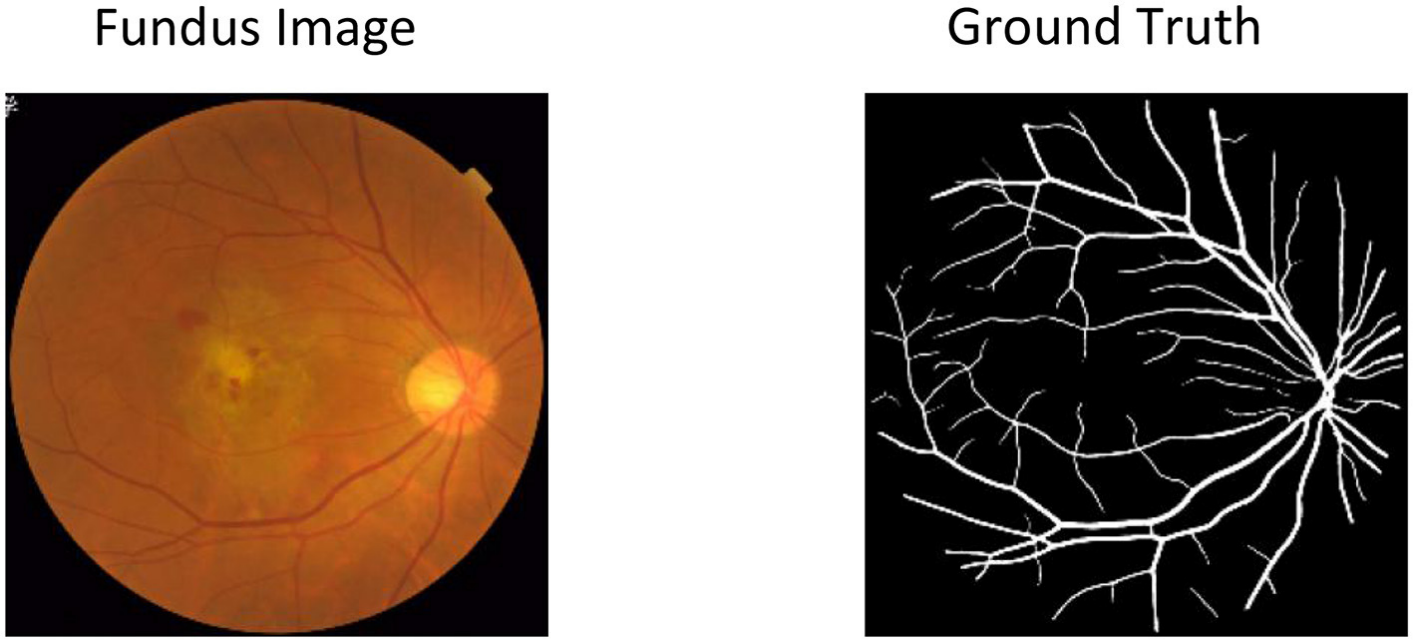
Sample retinal fundus image for vessel segmentation and hypertensive retinopathy quantification. The yellow areas in Ground Truth represent the retinal vessel area that needs to be segmented for disease analysis.

The retinal vascular system shares commonalities in structural, functional, and embryological aspects with the vascular systems of the heart, brain, and kidneys (5–9). Compared to other microvascular territories, the distinctive attributes of the retinal microcirculation enable relatively straightforward detection of localized HMOD (5, 9). Its capacity to offer a non-invasive and uncomplicated diagnostic tool positions retinal visualization as the simplest means of elucidating the microcirculatory system. In hypertensive patients, retinal microvasculature gives insight into the wellbeing of the heart, kidneys, and brain (5, 10, 11). Early detection of HT-mediated retinal changes indirectly mirrors the vascular status of these organs, facilitating refined evaluation of cardiovascular risk stratification, timely interventions, and improved prognostication, thereby holding substantial clinical significance. Traditional clinical methodologies for diagnosing HT-mediated retinal alterations, while reliant on the proficiency of ophthalmic professionals, often demand considerable time and specialized expertise (12). Figure 1 presents a sample fundus image, demonstrating the complexity of the retinal vasculature and image intensity variation. However, integrating AI-based models in ophthalmology holds promising prospects for revolutionizing this paradigm. Leveraging machine learning algorithms and deep neural networks, AI-enabled diagnostic tools have demonstrated the potential to expedite and enhance the assessment of HT-related retinal vessel changes (13–17). These AI models learn from extensive datasets of annotated medical images, swiftly recognizing subtle retinal anomalies that might elude human detection. By automating the analysis and interpretation of retinal images, AI-based systems offer the prospect of reducing diagnostic timeframes, improving accuracy, and potentially mitigating the need for extensive human oversight. In this work, we proposed a heterogeneous features cross-attention neural network to tackle the retinal vessel segmentation task with color fundus images.

## 2 Related work

Segmenting blood vessels in retinal color fundus images plays a pivotal role in the diagnostic process of hypertensive retinopathy. Over the years, researchers have explored computer-assisted methodologies to tackle this task. For instance, Annunziata and Trucco (18) introduced a novel curvature segmentation technique leveraging an accelerating filter bank implemented via a speed-up convolutional sparse coding filter learning approach. Their method employs a warm initialization strategy, kickstarted by meticulously crafted filters. These filters are adept at capturing the visual characteristics of curvilinear structures, subsequently fine-tuned through convolutional sparse coding. Similarly, Marín et al. (19) delved into the realm of hand-crafted feature learning methods, harnessing gray-level and moment invariant-based features for vessel segmentation. However, despite the efficacy of such techniques, the manual crafting of filters is inherently time-intensive and prone to biases, necessitating a shift toward more automated and data-driven approaches in this domain.

Deep learning techniques based on data analysis have demonstrated superior performance to conventional retinal vessel segmentation approaches (18–20). For instance, Maninis et al. (21) developed a method wherein feature maps derived from a side output layer contributed to vessel and optic disc segmentation. Along a similar line, Oliveira et al. (22) combined the benefits of stationary wavelet transform's multi-scale analysis with a multi-scale full convolutional neural network, resulting in a technique adept at accommodating variations in the width and orientation of retinal vessel structures. In terms of exploiting the advance of the Unit structure, there are previous methods that achieved promising performance. For example, Yan et al. (23) implemented a joint loss function in U-Net, comprising two components responsible for pixel-wise and segment-level losses, aiming to enhance the model's ability to balance segmentation between thicker and thinner vessels. Mou et al. (24) embedded dense dilated convolutional blocks between encoder and decoder cells at corresponding levels of a U-shaped network, employing a regularized walk algorithm for post-processing model predictions. Similarly, Wang et al. (25) proposed a Dual U-Net with two encoders: one focused on spatial information extraction and the other on context information. They introduced a novel module to merge information from both paths.

Despite the proficiency of existing deep learning methodologies in segmenting thicker vessels, there remains a challenge in combining heterogeneous features from different stages of the deep learning models via Transformers and CNN models. Generally, improving deep learning-based techniques for vessel segmentation can be approached from various angles, including multi-stage feature fusion and optimization of loss functions. This work proposes a heterogeneous feature cross-attention neural network to address the above challenge.

## 3 Materials and methods

### 3.1 Heterogeneous features cross-attention neural network

**Figure 2:**
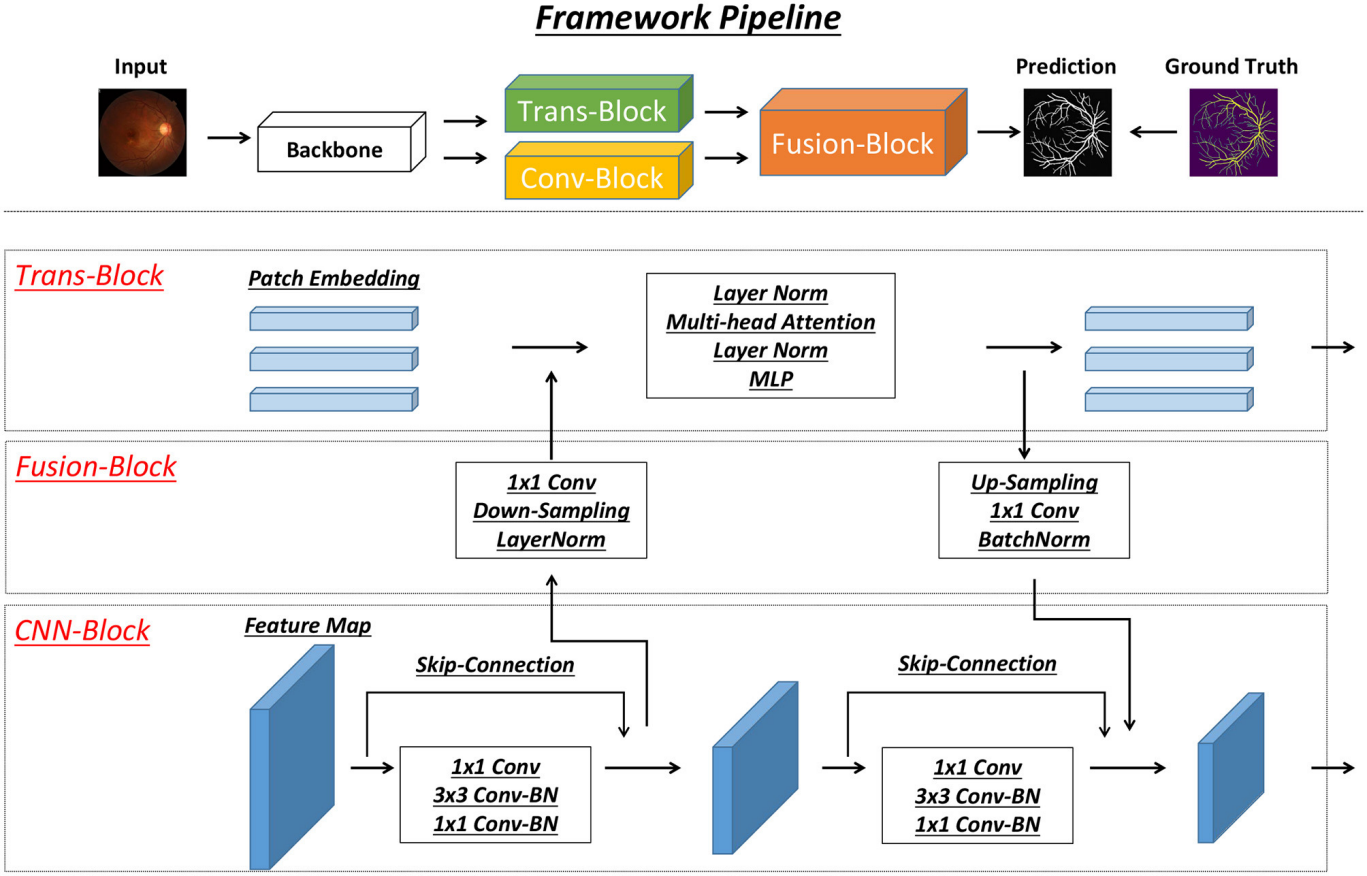
Figure of our proposed model structure. Our model contains three modules, including Trans-Block, CNN-Block and Fusion-Block. The detailed structure of each module is shown in the figure.

A detailed model structure overview is shown in Figure 2. In detail, two brunches of feature extraction modules are proposed to extract heterogeneous features from different stages of the backbone network. In detail, there is CNN-based (Conv-Block) and transformer-based (Trans-Block) brunch, which focus on local semantic and long-range spatial information. Those two features' information are both important for the vessel segmentation task.

The interaction between the two branches is used as a cross-attention module to emphasize the essential heterogeneous (semantic and spatial) features. It is used as the main structure to facilitate the interaction and integration of local and long-range global features. Drawing inspiration from the work by Peng et al. (26), the intersecting network architecture within our model ensures that both Conv-Block and Trans-Block can concurrently learn features derived from the preceding Conv-Block and Trans-Block, respectively.

#### 3.1.1 CNN blocks

In the structure depicted in Figure 2, the CNN branch adopts a hierarchical structure, leading to a reduction in the resolution of feature maps as the network depth increases and the channel count expands. Each phase of this structure consists of several convolution blocks, each housing multiple bottlenecks. These bottlenecks, in accordance with the ResNet framework (27), comprise a sequence involving down-projection, spatial convolution, up-projection, and a residual connection to maintain information flow within the block. Distinctly, visual transformers (28) condense an image patch into a vector in one step, which unfortunately leads to the loss of localized details. Conversely, in CNNs, the convolutional kernels operate on feature maps, overlapping to retain intricate local features. Consequently, the CNN branch ensures a sequential provision of localized feature intricacies to benefit the transformer branch.

#### 3.1.2 Transformer blocks

In line with the approach introduced in ViT (28), this segment consists of N sequential transformer blocks, as showcased in Figure 2. Each transformer block combines a multi-head self-attention module with an MLP block, encompassing an up-projection fully connected layer and a down-projection fully connected layer. Throughout this structure, LayerNorms (29) are applied before each layer, and residual connections are integrated into both the self-attention layer and the MLP block. For tokenization purposes, the feature maps generated by the backbone module are compressed into 16 × 16 patch embeddings without overlap. This compression is achieved using a linear projection layer, implemented via a 3 × 3 convolution with a stride of 1. Notably, considering that the CNN branch (3 × 3 convolution) encodes both local features and spatial location information, the necessity for positional embeddings diminishes. This strategic adaptation results in an improved image resolution, advantageous for subsequent tasks related to vision.

#### 3.1.3 Feature fusion blocks

Aligning the feature maps derived from the CNN branch with the patch embeddings within the transformer branch poses a significant challenge. To tackle this, we introduce the feature fusion block, aiming to continuously and interactively integrate local features with global representations. The substantial difference in dimensionalities between the CNN and transformer features is noteworthy. While CNN feature maps are characterized by dimensions *C*×*H*×*W* (representing channels, height, and width, respectively), patch embeddings assume a shape of (*L*+1) × *J*, where *L*, 1, and *J* denote the count of image patches, class token, and embedding dimensions, respectively. To reconcile these disparities, feature maps transmitted to the transformer branch undergo an initial 1 × 1 convolution to align their channel numbers with the patch embeddings. Subsequently, a down-sampling module (depicted in Figure 2) aligns spatial dimensions, following which the feature maps are amalgamated with patch embeddings, as portrayed in Figure 2. Upon feedback from the transformer to the CNN branch, the patch embeddings necessitate up-sampling (as illustrated in Figure 2) to match the spatial scale. Following this, aligning the channel dimension with that of the CNN feature maps through a 1 × 1 convolution is performed, integrating these adjusted embeddings into the feature maps. Furthermore, LayerNorm and BatchNorm modules are employed to regularize the features. Moreover, a significant semantic disparity arises between feature maps and patch embeddings. While feature maps stem from local convolutional operators, patch embeddings arise from global self-attention mechanisms. Consequently, the feature fusion block is incorporated into each block (excluding the initial one) to bridge this semantic gap progressively.

### 3.2 Experiments

#### 3.2.1 Datasets

Four public datasets, *DRIVE* (30), *CHASEDB1* (31), *STARE* (32), and *HRF* (33), were used in our experiments. The images of these datasets were captured by different devices and with different image sizes. A detailed description of each dataset is elaborated below:

1) *DRIVE* dataset: the dataset known as *DRIVE* comprises 40 pairs of fundus images accompanied by their respective labels for vessel segmentation. Each image within this dataset measures 565 × 584 pixels. Furthermore, the dataset has been partitioned into distinct training and test sets, encompassing 20 pairs of images and corresponding labels within each set. Notably, in the test set, every image has undergone labeling by two medical professionals. Typically, the initial label is considered the reference standard (ground truth), while the second label serves as a human observation used to assess accuracy.
2) *CHASEDB1* dataset: the CHASEDB1 dataset encompasses a collection of 28 images, comprising samples from both the left and right eyes, with each image possessing dimensions of 999 × 960 pixels. Past investigations have specifically delineated the dataset's utilization, designating a distinct partition for training and testing purposes. According to prior scholarly research (31), a selection strategy has been employed, with the final eight images demarcated for evaluation as testing samples, while the preceding images have been earmarked for utilization as training samples. This segmentation strategy in the dataset facilitates a structured approach for model training and evaluation, enabling a systematic analysis of algorithm performance on separate subsets of images to ensure robustness and generalizability in vessel segmentation tasks.
3) *STARE* dataset: each image within the *STARE* dataset measures 700 × 605 pixels. This dataset comprises 20 color fundus images without a predefined division into training and test sets. Previous studies have employed two common schemes for test set allocation to assess method performance. One approach involves assigning 10 images to the training set and the remaining 10 to the test set. Alternatively, the Leave-One-Out method has been utilized, wherein each image successively serves as the test set while the remaining images form the training set for evaluation purposes in different iterations.
4) *HRF* dataset: the HRF dataset comprises 45 fundus images with a resolution of 3,504 × 2,336 pixels. From this dataset, 15 images from are allocated to the training set, while the remaining 30 images constitute the test set. To mitigate computational expenses, both the images and their corresponding labels are downsampled twice, as noted in (34).

#### 3.2.2 Loss functions

Commonly utilized region-based losses, like Dice loss (35), often result in highly precise segmentation. However, they tend to disregard the intricate vessel shapes due to a multitude of pixels outside the target area, overshadowing the significance of those delineating the vessel (36–40). This oversight may contribute to relatively imprecise retinal vessel segmentation and, consequently, inaccurate quantification of hypertensive retinopathy. In response, we incorporated the TopK loss (Equation 1) (41, 42) to emphasize the retinal vessels during the training process specifically. When objects exhibit sizes that are not notably smaller in comparison to the convolutional neural network's (CNN) receptive field, the vessel emerges as the most variable component within the prediction, displaying the least certainty; thus, the loss within the vessel region tends to be the highest among the predictions (43). Building upon these observations and rationale, the TopK loss is formulated as follows:


(1) $$\begin{array}{l} L_{TopK}=-\frac{1}{N}\underset{i\in K}{\sum }g_{i}\log s_{i} \end{array}$$

where *g*_*i*_ is the ground truth of pixel *i*, *s*_*i*_ is the corresponding predicted probability, and *K* is the set of the *k%* pixels with the lowest prediction accuracy. While sole vessel-focused loss often causes training instability (44), region-based loss, such as Dice loss (Equation 2) (35), is needed at the early stage of the training. We represent Dice loss as follows:


(2) $$\begin{array}{l} L_{Dice}=1-\frac{2|V_{s}\cap V_{g}|}{\left|V_{s}|+|V_{g}\right|} \end{array}$$

where *V*_*g*_ is the ground truth label and *V*_*s*_ is the prediction result of segmentation. We coupled TopK with region-based Dice loss as our final loss function (Equation 3) for the retinal vessel segmentation.


(3) $$\begin{array}{l} L=L_{TopK}+L_{Dice} \end{array}$$

#### 3.2.3 Experimental setting

To enrich the dataset, we introduce random rotations on the fly to the input images in the training dataset, applied to both segmentation tasks. Specifically, these rotations span from –20 to 20 degrees. Additionally, 10% of the training dataset is randomly chosen to serve as the validation dataset. The proposed network was implemented utilizing the PyTorch Library and executed on the Nvidia GeForce TITAN Xp GPU. Throughout the training phase, we employed the AdamW optimizer to fine-tune the deep model. To ensure effective training, a gradually decreasing learning rate was adopted, commencing at 0.0001, alongside a momentum parameter set at 0.9. For each iteration, a random patch of size 118 × 118 from the image was selected for training purposes, with a specified batch size of 16. A backbone of ResNet50 (27) is used in this work.

#### 3.2.4 Evaluation metrics

The model's output is represented as a probability map, assigning to each pixel the probability of being associated with the vessel class. Throughout the experiments, a probability threshold of 0.5 was employed to yield the results. To comprehensively assess the efficacy of our proposed framework during the testing phase, the subsequent metrics will be computed:

- Acc (accuracy) = (TP + TN) / (TP + TN + FP + FN),
- SE (sensitivity) = TP / (TP + FN),
- SP (specificity) = TN / (TN + FP)
- F1 (F1 score) = (2 × TP) / (2 × TP + FP + FN)
- AUROC = area under the receiver operating characteristic curve.

In this context, the correct classification of a vessel pixel is categorized as a true positive (TP), while misclassification is identified as a false positive (FP). Correspondingly, accurate classification of a non-vessel pixel is considered a true negative (TN), whereas misclassification is denoted as a false negative (FN).

### 3.3 Compared methods

We compared our approach to other classic and state-of-the-art models that have achieved promising performance on different medical image segmentation tasks. All of the experiments are conducted under the same experimental setting. The compared methods are briefly introduced below:

- Unet (45): Unet is a CNN architecture used for image segmentation tasks. Its U-shaped design includes an encoder (contracting path) for feature extraction and a symmetric decoder (expansive path) for generating segmented outputs. The network uses skip connections to preserve fine details and context, making it effective for tasks like biomedical image segmentation.
- Unet++ (46): Unet++ is an advanced version of the U-Net architecture designed for image segmentation tasks. It improves upon U-Net by introducing nested skip connections and aggregation pathways, allowing better multi-scale feature integration and context aggregation. This enhancement leads to more accurate and precise segmentation results compared to the original U-Net model.
- Swin-Transformer (47): Swin-Transformer is a hierarchical vision transformer (28) structure. It uses shifted windows to process image patches hierarchically, allowing for improved global context understanding. This architecture has demonstrated competitive segmentation performance with efficient computation.
- AttenUnet (48): The AttenUnet enhances the traditional U-Net architecture that integrates attention mechanisms. These mechanisms enable the network to focus on important image features during segmentation tasks. It improves accuracy by refining object delineation and suppressing irrelevant information. This variant is particularly effective in tasks like medical image segmentation, where precise localization of structures is essential.
- TransUnet (49): TransUNet is a proposed architecture to improve medical image segmentation, addressing limitations seen in the widely used U-Net model. It combines the strengths of Transformers' global self-attention with U-Net's precise localization abilities. The Transformer part encodes image patches from a CNN feature map to capture global context, while the decoder integrates this with high-resolution feature maps for accurate localization.

## 4 Results

### 4.1 Vessel segmentation performance

**Figure 3:**
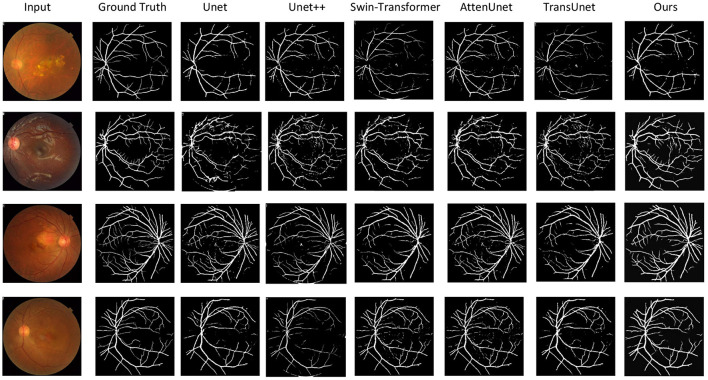
Qualitative results of the vessel segmentation. We compare our model with Unet (45), Unet++ (46), Swin-Transformer (47), AttenUnet (48), TransUnet (49). Our method can produce more accurate segmentation results than the other methods compared with the ground truth.

Figure 3 illustrates qualitative comparison with other compared methods on the test dataset. Tables 1–4 shows the quantitative performance of *Ours* and other methods on four different datasets, respectively.

**Table 1:** Quantitative results comparison between our methods and other compared state-of-the-art methods on *DRIVE* dataset.

**Methods**	***Acc***	***SE***	***SP***	***F1***	***AUROC***
*Unet*	90.1 (89.1, 90.8)	76.5 (74.2, 78.1)	97.7 (95.8, 99.1)	80.3 (78.3, 82.3)	97.2 (95.0, 98.0)
*Unet++*	91.3 (90.4, 92.7)	79.2 (78.0, 80.6)	97.9 (95.2, 99.0)	81.0 (79.2, 82.5)	97.1 (95.8, 99.0)
*Swin-Transformer*	92.3 (91.5, 92.9)	79.0 (77.9, 80.6)	98.1 (96.4, 99.2)	82.0 (81.0, 84.0)	97.6 (96.1, 98.3)
*AttenUnet*	92.1 (91.3, 93.2)	80.0 (78.3, 82.0)	98.3 (96.1, 99.5)	80.4 (78.5, 82.1)	97.4 (96.2, 98.6)
*TransUnet*	91.8 (91.2, 93.0)	80.3 (79.1, 81.3)	98.3 (97.2, 99.6)	80.1 (78.8, 80.9)	97.3 (96.4, 99.0)
*Ours*	**93.8** (92.9, 94.8)	**81.0** (80.2, 82.6)	**98.5** (96.7, 99.1)	**83.3** (78.8, 82.1)	**97.9** (96.2, 98.8)

Performance is reported with *Acc, SE, SP, F1* and *AUROC*. 95% confidence interval is presented in the bracket. The best performance is highlighted in bold.

**Table 2:** Quantitative results comparison between our methods and other compared state-of-the-art methods on *CHASEDB1* dataset.

**Methods**	***Acc***	***SE***	***SP***	***F1***	***AUROC***
*Unet*	91.2 (89.8, 92.3)	60.3 (58.2, 61.4)	97.1 (96.4, 97.9)	79.7 (76.9, 81.0)	97.7 (96.6, 98.2)
*Unet++*	91.6 (89.8, 93.2)	63.0 (61.2, 65.0)	97.3 (95.5, 98.3)	80.1 (78.5, 82.1)	97.7 (96.2, 98.3)
*Swin-Transformer*	92.3(91.0, 94.1)	62.9 (61.4, 64.0)	97.8 (96.2, 98.5)	80.3 (78.7, 81.7)	97.9 (96.2, 98.8)
*AttenUnet*	92.4 (91.0, 94.2)	67.7 (65.5, 68.3)	97.7 (96.2, 98.4)	79.9 (77.4, 80.6)	97.8 (97.0, 98.5)
*TransUnet*	92.6 (90.2, 94.4)	66.1 (64.6, 67.7)	98.0 (96.7, 99.0)	80.4 (78.9, 82.1)	98.2 (96.3, 99.9)
*Ours*	**93.7** (91.7, 95.2)	**69.0** (67.4, 70.5)	**98.9** (97.2, 99.3)	**81.6** (81.0, 93.0)	**98.9** (98.1, 99.3)

Performance is reported with *Acc, SE, SP, F1* and *AUROC*. 95% confidence interval is presented in the bracket. The best performance is highlighted in bold.

**Table 3:** Quantitative results comparison between our methods and other compared state-of-the-art methods on *STARE* dataset.

**Methods**	***Acc***	***SE***	***SP***	***F1***	***AUROC***
*Unet*	93.3 (91.7, 95.2)	80.8 (78.7, 81.8)	98.1 (97.1, 99.0)	84.3 (82.2, 86.3)	98.1 (97.0, 99.0)
*Unet++*	94.2 (92.5, 96.0)	82.6 (81.6, 83.1)	98.0 (96.4, 99.0)	84.5 (83.7, 85.2)	98.3 (97.1, 99.2)
*Swin-Transformer*	93.9 (92.8, 94.7)	83.0 (82.0, 84.2)	98.2 (96.9, 99.1)	84.1 (82.5, 86.2)	98.5 (97.4, 99.3)
*AttenUnet*	93.6 (92.7, 94.7)	82.9 (81.7, 84.2)	98.6 (96.2, 99.3)	84.6 (82.9, 86.3)	98.6 (96.7, 99.5)
*TransUnet*	93.4 (91.9, 94.7)	83.2 (81.6, 85.0)	98.7 (96.6, 99.4)	85.2 (83.7, 86.9)	98.1 (97.2, 99.1)
*Ours*	**94.8** (92.9, 95.6)	**84.2** (82.6, 86.1)	**99.2** (97.7, 99.4)	**86.6** (85.9, 87.4)	**99.3** (98.4, 99.7)

Performance is reported with *Acc, SE, SP, F1* and *AUROC*. 95% confidence interval is presented in the bracket. The best performance is highlighted in bold.

**Table 4:** Quantitative results comparison between our methods and other compared state-of-the-art methods on *HRF* dataset.

**Methods**	***Acc***	***SE***	***SP***	***F1***	***AUROC***
*Unet*	94.4 (92.3, 96.0)	77.7 (75.8, 79.0)	95.1 (93.8, 96.7)	78.6 (76.9, 79.1)	97.2 (96.0, 98.0)
*Unet++*	94.8 (92.8, 96.2)	78.9 (78.0, 79.6)	95.1 (93.8, 96.4)	79.3 (78.7, 80.5)	97.3 (96.1, 98.3)
*Swin-Transformer*	94.6 (92.9, 96.0)	79.1 (77.9, 80.5)	94.4 (92.7, 96.0)	79.5 (77.7, 80.6)	97.8 (96.2, 98.6)
*AttenUnet*	95.8 (93.9, 96.9)	77.6 (75.8, 79.1)	94.6 (93.9, 95.4)	78.8 (76.9, 79.5)	98.2 (97.0, 99.0)
*TransUnet*	95.3 (94.2, 96.3)	78.6 (77.4, 79.8)	94.7 (92.9, 96.3)	78.9 (77.0, 79.9)	98.3 (97.2, 99.1)
*Ours*	**96.2** (95.0, 97.1)	**79.9** (78.0, 81.0)	**94.9** (92.8, 96.0)	**79.9** (77.9, 81.2)	**98.8** (97.9, 99.3)

Performance is reported with *Acc, SE, SP, F1* and *AUROC*. 95% confidence interval is presented in the bracket. The best performance is highlighted in bold.

Our proposed method can outperform other compared methods on *DRIVE, CHASEDB1, STARE*, and *HRF* datasets, respectively. In detail, *Ours* achieved 83.3% *F1* on *DRIVE* dataset, which outperformed *Unet* (45) by 3.6%, outperformed *Swin-Transformer* (47) by 1.6% and outperformed *TransUnet* (49) by 4.0%. *Ours* achieved 81.6% *F1* on *CHASEDB1* dataset, which outperformed *Unet++* (46) by 1.9%, outperformed *AttenUnet* (48) by 2.1% and outperformed *TransUnet* (49) by 1.5%. *Ours* achieved 86.6% *F1* on *STARE* dataset, which outperformed *Unet* (45) by 2.7%, outperformed *AttenUnet* (48) by 2.4% and outperformed *TransUnet* (49) by 1.6%. *Ours* achieved 79.9% *F1* on *HRF* dataset, which outperformed *Unet++* (46) by 0.8%, outperformed *Swin-Transformer* (47) by 0.5% and outperformed *TransUnet* (49) by 1.3%. Notably, *Swin-Transformer* (47) and *TransUnet* (49) belong to the transformer-based model structure, which demonstrates a superior performance on many tasks. However, in this work, the limited data size is one of the leading reasons for the relatively low performance of those datasets. Another reason could be the task's own nature of vessel segmentation, where more local information is needed rather than the long-range relationship between pixels. Thus, given two brunches with transformer and CNN structures and fusion modules, our proposed model can simultaneously tackle both the local semantic information and long-range spatial information for the segmentation task.

Figure 3 shows the qualitative comparison between ours and other compared methods. It demonstrated that our proposed methods can segment the vessels more accurately. This is important for vessel segmentation tasks and hypertensive retinopathy quantification with more accurate vessel area calculation.

### 4.2 Ablation study

#### 4.2.1 Ablation study on loss functions

We did ablation study experiments on loss functions. We maintain the same model structure and only change the loss functions. In detail, we remove Dice loss and TopK loss, respectively, to evaluate their respective contribution to the performance of the proposed models. Furthermore, we replace TopK loss with a cross-entropy loss to validate the effectiveness of TopK loss in the segmentation task. Table 5 demonstrates that Dice Loss can lead to a 6.2% *F1* and *TopK* loss can lead to a 2.9% *F1* performance. On the other hand, Dice loss can lead to 15.5% *SE* performance, and *TopK* loss can lead to a 2.8% *SE* performance on *Drive* dataset. Additionally, compared with cross-entropy loss, the TopK loss could lead to a 1.5% *F1* improvement and 2.3% *SE* improvement. Each loss function can boost the model's performance in different evaluation metrics. This demonstrated that the adopted loss function can both contribute to the learning process and benefit the vessel segmentation performance.

**Table 5:** Quantitative ablation study results of the loss function on DRIVE dataset.

**Methods**	***Acc***	***SE***	***SP***	***F1***	***AUROC***
*w/o Dice loss*	86.4 (85.0, 88.0)	70.1 (68.2, 72.5)	94.4 (92.3, 96.0)	75.6 (74.1, 76.2)	94.5 (92.8, 95.6)
*w/o TopK loss*	88.9 (87.3, 89.6)	78.8 (76.9, 80.3)	96.0 (94.2, 97.2)	78.0 (77.0, 79.2)	96.3 (94.8, 97.7)
*w/ Cross-entropy loss*	90.3 (89.6, 91.0)	79.2 (78.5, 80.0)	96.9 (95.8, 97.4)	79.1 (78.0, 80.2)	96.9 (95.8, 97.5)
*Ours*	**93.8** (92.9, 94.8)	**81.0** (80.2, 82.6)	**98.5** (96.7, 99.1)	**80.3** (78.8, 82.1)	**97.9** (96.2, 98.8)

Performance is reported with *Acc, SE, SP, F1* and *AUROC*. 95% confidence interval is presented in the bracket. The best performance is highlighted in bold.

#### 4.2.2 Ablation study on the models' components

We did ablation study experiments on the model's components. In detail, we maintain the same model structure and only change the models' structure by removing different modules, including *Trans-Block, CNN-Block* and *Fusion-Block*, respectively. In detail, we remove each of those three modules, respectively, to evaluate their respective contribution to the performance of the proposed models. Table 6 demonstrates that *Trans-Block* can lead to a 10% *F1, CNN-Block* can lead to a 10.3% *F1* performance and *Fusion-Block* can lead to a 7.9% *F1* performance boost. On the other hand, *Trans-Block* can lead to a 3.3% *SE* performance, *CNN-Block* can lead to a 2.3% *SE* performance, and *Fusion-Block* can lead to an 0.9% *SE* performance on *Drive* dataset. Each module can boost the model's performance in different evaluation metrics. This demonstrated that the proposed modules can all contribute to the learning process and benefit the vessel segmentation performance.

**Table 6:** Quantitative ablation study results of the model's components on DRIVE dataset.

**Methods**	***Acc***	***SE***	***SP***	***F1***	***AUROC***
*w/o Trans-Block*	88.9 (87.6, 89.5)	78.4 (76.8, 79.3)	92.1 (91.2, 92.9)	73.0 (71.5, 74.6)	95.2 (93.7, 96.6)
*w/o CNN-Block*	89.1 (87.9, 90.8)	79.2 (78.2, 80.6)	92.3 (91.4, 92.9)	72.8 (71.6, 73.5)	95.3 (93.8, 96.6)
*w/o Fusion-Block*	91.2 (89.9, 92.3)	80.3 (78.8, 81.6)	93.1 (92.1, 94.4)	74.4 (72.6, 76.6)	96.3 (95.8, 96.7)
*Ours*	**93.8** (92.9, 94.8)	**81.0** (80.2, 82.6)	**98.5** (96.7, 99.1)	**80.3** (78.8, 82.1)	**97.9** (96.2, 98.8)

Performance is reported with *Acc, SE, SP, F1* and *AUROC*. 95% confidence interval is presented in the bracket. The best performance is highlighted in bold.

## 5 Hypertensive retinopathy quantification

The proposed method has demonstrated a promising retinal vessel segmentation performance on different datasets and benchmarks. Additionally, precise segmentation of retinal vessels plays a vital role in hypertensive retinopathy detection, whereas manual segmentation tends to be cumbersome and time-consuming (50). The model proposed can generate a binary mask distinguishing vessel pixels as one and background pixels as zero. This mask effectively quantifies the total count of vessel pixels within each mask. The ratio (*R*_*vessel*_) between the count of vessel pixels and non-vessel pixels is defined as follows:


(4) $$\begin{array}{l} R_{vessel}=\frac{N_{v}}{N_{non}-N_{v}}, \end{array}$$

where *N*_*v*_ represents the count of vessel pixels, and *N*_*non*_ denotes the count of non-vessel pixels. The ratio *R*_*vessel*_ (Equation 4) serves as a valuable metric in identifying hypertensive retinopathy within fundus images. Hypertensive retinopathy leads to vascular constriction (51, 52), resulting in a decrease in the count of vessel pixels (*R*_*vessel*_).

Detection of hypertensive retinopathy, characterized by vascular constriction, involves assessing changes in *R*_*vessel*_ across sequential examinations. Increases or decreases in *R*_*vessel*_ indicate the occurrence or progression of hypertensive retinopathy, respectively. Hence, our proposed methods offer a straightforward approach for detecting hypertensive retinopathy.

In the future, with increased datasets comprising fundus images from hypertensive and healthy patients, we can further analyze vessel changes within these images. In real-world clinical practice, comparing the *R*_*vessel*_ obtained from consecutive visits can serve as a diagnostic tool. Additionally, the detection of newly formed vessels can be achieved by subtracting images from successive visits post-segmentation. This approach enables the identification and tracking of changes in vasculature over time, offering potential insights for clinical assessment and monitoring.

## 6 Limitation and future works

While our deep learning method has shown promising results in the challenging tasks of retinal vessel segmentation and hypertensive retinopathy quantification, it's important to acknowledge the nuanced landscape of limitations accompanying such endeavors. One notable factor is the inherent variability present in medical imaging datasets. Our model's performance could be influenced by factors such as variations in image quality and disease severity across different datasets. Moreover, despite achieving commendable results overall, there are instances where the model might struggle to accurately delineate intricate vascular structures or detect subtle manifestations of hypertensive retinopathy. This suggests the need for further exploration and refinement of our approach.

In future research, attention could be directed toward enhancing the model's robustness and adaptability to diverse imaging conditions and patient populations. Techniques such as advanced data augmentation and domain adaptation strategies could prove instrumental in achieving this goal. Additionally, integrating complementary sources of information, such as clinical metadata or genetic markers, holds promise for enriching the predictive capabilities of our model and enhancing its clinical relevance. Furthermore, the pursuit of interpretability and explainability remains paramount. Providing clinicians with insights into how the model arrives at its predictions can foster trust and facilitate its integration into real-world clinical workflows. However, this pursuit must be balanced with ethical considerations, particularly concerning patient privacy, algorithmic bias, and the potential consequences of automated decision-making in healthcare settings. By addressing these multifaceted challenges, we can pave the way for more effective and responsible deployment of deep learning technologies in ophthalmology and beyond.

## 7 Conclusion

We have proposed a novel and comprehensive framework for retinal vessel segmentation and hypertensive retinopathy quantification. It takes advantage of heterogeneous feature cross-attention with the help of local emphasis CNN and long-range emphasis transformer structure with a fusion module to aggregate the information. Our experiments on four large-scale datasets have demonstrated that our framework can simultaneously conduct accurate segmentation and potential hypertensive retinopathy quantification performance.

## Data availability statement

The original contributions presented in the study are included in the article/supplementary material, further inquiries can be directed to the corresponding author.

## Author contributions

XL: Writing – review & editing, Writing – original draft, Visualization, Validation, Software, Methodology, Formal analysis, Data curation. HT: Writing – review & editing, Writing – original draft, Visualization, Validation, Resources, Formal analysis, Conceptualization. WW: Writing – review & editing, Writing – original draft, Visualization, Validation, Software, Methodology. ZC: Writing – review & editing, Writing – original draft, Supervision, Resources, Project administration, Funding acquisition, Conceptualization.
